# Campbell title registrations to date

**DOI:** 10.1002/cl2.1356

**Published:** 2023-09-18

Details of new titles for systematic reviews or evidence and gap maps that have been accepted by the Editor of a Campbell Coordinating Group are published in each issue of the journal. If you would like to receive a copy of the approved title registration form, please send an email to the Managing Editor of the relevant Coordinating Group.

**AGEING**

Understanding practices and support needs of family caregivers of people with dementia in Africa: A scoping review

Oluwagbemiga Oyinlola, Tamara Sussman, Anthony Iwuagwu

14 June 2023

Barriers to and facilitators of the implementation of nonpharmacological interventions for behaviors and psychological symptoms of dementia in residential aged care homes: A systematic review of qualitative studies

Hunduma Ayeno, Gizat Kassie, Mustafa Atee, Tuan Nguyen

23 June 2023

**INTERNATIONAL DEVELOPMENT**

Barriers and facilitators of community participation for improving maternal and child health utilisation and outcomes in low‐and‐middle‐income countries: A mixed‐method systematic review Wapangjungla Longchar, Sibasis Hense, Denny John

14 June 2023

Digital tools and job aids used by and for community health workers in low‐ and middle‐income countries to improve health outcomes and increase effectiveness: An evidence and gap map

William Philbrick, Jacob Milnor, Zeus Aranda, Feven Mekuria, Perpetua Mbachu, Brian Ssennoga, Patricia Mechael

14 August 2023

**CHILDREN AND YOUNG PERSONS WELLBEING**

Nutritional interventions in the management of childhood/youth aggression and other externalizing behaviours: A systematic review and meta‐analysis

Barna Konkolÿ Thege

16 June 2023

Examining the value of theatre for young people: A global systematic review and evidence gap map

Kelly Freebody, Michael Anderson, Eliza Oliver

11 August 2023

**KNOWLEDGE TRANSLATION AND IMPLEMENTATION**

Electronic hand hygiene monitoring for health care settings: An evidence and gap map

Donna Moralejo, Jane Chudleigh, Dinah Gould, Edward Pursssell, Nicholas Drey, Clare Hawker

27 July 2023

The application of information and communication technologies in physical activity interventions for breast cancer: A scoping review

Xin Chen, Maaz Imam, Yutong Yi, JJ Pionke, Lixcy Vega, Anna Arthur, Jessie Chin, Chungyi Chiu

27 July 2023

**EDUCATION**

Service learning interventions effectiveness in tertiary education: A systematic review

Patricia Ayllón Salas, Mirian Hervás Torres, Francisco Fernández Martín, Marta Pellegrini, Jose Arco Tirado, Andrew Furco

4 June 2023

Experiences and knowledge of racism during medical school: A systematic review

Karan Varshney, Mariyah Hoosenally, Jacqueline Rusnak, Devni Nagodavithana, Charlie Ho 5 July 2023

Opportunities and challenges of undergraduate health professional students' virtual clinical skills assessment during the Covid‐19 pandemic: A systematic review

Marema Jebessa Kumsa, Girma Awaki Feyera, Daniel Desta Dolisso, Tefera Tadesse

6 July 2023

Learner‐educator co‐creation of student assessment in health professional education courses: A scoping review

Laura Killam, Rylan Egan, Christina Godfrey, Amanda Ross‐White, Pilar Camargo‐Plazas, Mercedes Lock, Marian Luctkar‐Flude

28 July 2023

Prevention intervention and compensation programs to tackle school dropout: An evidence and gap map

Marta Pellegrini, Carmen Pannone, Daniela Fadda, Amanda Neitzel, Laura Francesca Scalas, Giuliano Vivanet

28 July 2023

